# Antioxidant and Antimicrobial Activity of Honey Bee Products—2nd Edition

**DOI:** 10.3390/antiox15010099

**Published:** 2026-01-13

**Authors:** Ivana Tlak Gajger, Josipa Vlainić

**Affiliations:** 1 Department for Biology and Pathology of Fish and Bees, NRL for Honeybee Diseases, Faculty of Veterinary Medicine, University of Zagreb, Heinzelova 55, 10000 Zagreb, Croatia; 2 Institute Ruđer Bošković, Bijenička Cesta 54, 10000 Zagreb, Croatia; josipa.vlainic@irb.hr

Antioxidants have acquired a central position in modern nutrition and preventive medicine, and honey bee products are increasingly recognized as complex natural matrices that provide a rich and diverse source of these compounds [1]. Building on the success of the previous Special Issue “Antioxidant Activity of Honey Bee Products” (https://www.mdpi.com/journal/antioxidants/special_issues/Honey_Bee_Products, accessed on 31 October 2025), this new edition, “Antioxidant and Antimicrobial Activity of Honey Bee Products—2nd Edition” (https://www.mdpi.com/journal/antioxidants/special_issues/SP679NDK5I, accessed on 1 January 2026), broadens the focus by integrating antioxidant and antimicrobial properties and emphasizing their joint relevance for health promotion and disease prevention in an era dominated by chronic, inflammation- and oxidative stress-driven disorders [2].

Honey and its products, including propolis, honey bee pollen, honey bee bread, royal jelly, beeswax, and honey bee venom, are increasingly regarded not only as traditional remedies, but also as complex functional ingredients capable of modulating oxidative stress, inflammation and immune responses, primarily through a concerted action of their polyphenols, flavonoids, enzymes and bee-derived peptides such as defensin-1 [3,4,5]. Also, comparative studies of monofloral, multifloral, and honeydew honeys have shown that samples with higher total phenolic content exhibit stronger radical-scavenging capacity together with more pronounced antibacterial effects, in some cases approaching or rivaling the activity of benchmark honeys such as manuka and thus emerge as promising candidates for targeted nutritional and therapeutic applications [6,7].

An attractive feature of this Special Issue was the systematic comparison of antioxidant and antimicrobial properties across multiple honey bee products and their combinations, highlighting that pollen and honey bee bread, rich in phenolic acids such as p-coumaric and rosmarinic acid, can display notable antioxidant potential coupled with inhibition of clinically relevant bacterial strains and opportunistic fungi, supporting their positioning as “superfoods” with potential impact on chronic disease prevention. Equally compelling were the contributions that demonstrated synergistic effects in multi-component formulations. Mixtures of honey with polyphenol-rich plant extracts, as well as combinations of honey, royal jelly, and propolis, exhibited enhanced biochemical, histological, and immunohistochemical markers, protecting against oxidative and inflammatory damage. This illustrates the potential for bee-based interventions in complex pathophysiological conditions. A recurrent and conceptually important theme was the integration of detailed chemical characterization with functional outcomes, enabling the Special Issue content to move beyond simply cataloging antioxidant capacity and minimum inhibitory concentrations. By coupling advanced analytical techniques with a battery of in vitro antioxidant assays and antimicrobial tests against Gram-positive and Gram-negative bacteria, yeasts, and filamentous fungi, it was demonstrated that compositional patterns underlie both enhanced redox activity and antimicrobial performance. This multidimensional, mechanism-oriented perspective, spanning composition, processing effects, bioaccessibility, bioavailability, and biological efficacy across diverse honey bee products, provides this Special Issue a distinctive application-oriented profile as well as a strong scientific foundation for the development of standardized, evidence-based functional products from the hive [see the Contributions list].

This volume is organized into several interconnected sections that guide the reader from fundamental concepts to applied aspects. The first part focuses on the chemical composition, analytical characterization, and mechanistic understanding of antioxidant and antimicrobial activities of individual honey bee products. The second part brings together experimental and clinical studies that explore the impact of bee-derived antioxidants on biomarkers of oxidative stress, inflammation, and selected chronic diseases. The final chapters address technological, processing-related, and regulatory perspectives relevant to the development and standardization of functional products from the hive. This book is intended for a broad scientific and professional audience, including researchers in food and nutritional science, veterinary and human medicine, pharmacy, toxicology, and microbiology, as well as stakeholders from the food, nutraceutical, and pharmaceutical industries and regulatory bodies interested in evidence-based valorization of honey bee products.

This Special Issue features nine original articles and three reviews. The first notable contribution discusses a standardized bee-derived propolis extract, which represents a promising next-generation strategy for treating acne. This extract effectively targets pathogenic biofilms of *Cutibacterium acnes*, reducing its virulence while preserving the diversity of the skin microbiome and supporting antioxidant and anti-inflammatory responses in the epidermis (Contribution 1). In addition, process innovations such as ultrasound-assisted extraction are redefining how propolis bioactive compounds are obtained, with optimized ethanolic conditions yielding higher polyphenol and flavonoid levels, stronger antioxidant performance across multiple assays, and superior antimicrobial effects against foodborne Gram-positive pathogens, while preserving notable bioaccessibility after simulated digestion and thereby expanding the technological space for propolis-enriched health products (Contribution 2). According to an article about an integrated workflow that combines palynological examination with comprehensive physicochemical and phenolic–flavonoid profiling, along with extensive antibacterial and antifungal testing from a single propolis sample, now provides a resource-efficient method to connect the botanical origins of propolis with its bioactive potential. This approach aims to improve the development of standardized, evidence-based propolis preparations (Contribution 3). Advancements in formulation science now allow honey bee propolis to be transformed into stable, consumer-friendly delivery systems, expanding its health applications beyond just topical acne care. By microencapsulating ethanolic propolis extracts with plant and protein carriers and subsequent spray-drying, researchers have achieved improved solubility, thermal stability, and controlled release of phenolic bioactives, while successfully incorporating these microcapsules into functional gummies without loss of sensory quality, thereby opening avenues for complementary, ingestible formats of propolis-based interventions (Contribution 4).

In parallel, emerging work on kombucha beverages fortified with fermented bee-collected pollen highlights a complementary, ingestible route to harness bee-derived phenolics and phenylamides, enhancing antimicrobial functionality and reshaping the underlying polyphenol profile towards flavanol- and phenolic-acid-rich matrices that may synergize with propolis-based dermal care (Contribution 5). A long-term study on the storage of bee pollen highlights the importance of carefully controlling drying and freezing processes. This is crucial for preserving phenolic compounds, ascorbic acid, and in vitro antioxidant capacity, which help maintain its value as a nutritionally rich, polyfloral ingredient primarily composed of *Salix* and *Brassica napus* pollens (Contribution 6). Honey bee pollen’s remarkable nutritional and antioxidant potential is further underscored by work linking its palynological profile to phenolic richness and radical-scavenging capacity, with highly active monofloral Cistus- and Brassica-type pollens emerging as especially valuable polyphenol sources (Contribution 7). Most recently, cutting-edge chromatographic and effect-directed analyses have revealed N1,N5N10-tricaffeoylspermidine as a shared, highly bioactive marker in bee pollen and tree androecia from *Castanea*, *Salix*, and *Quercus* species, providing a new chemotaxonomic handle and highlighting xanthine oxidase inhibition as an additional mechanistic pathway through which well-characterized bee pollen preparations may contribute to disease-preventive nutraceutical concepts (Contribution 8).

Emerging in vivo data now extend the bee-product paradigm beyond propolis and pollen, showing that beeswax alcohol can outperform coenzyme Q10 in a zebrafish model of high-cholesterol and D-galactose burden by improving survival, dyslipidemia, redox balance, liver integrity, and cognition, thereby positioning structured beeswax lipids as promising candidates for future brain–liver axis-targeted nutraceuticals (Contribution 9).

At an ecological level, these insights highlight the role of pollen-derived polyphenols as crucial nutritional modulators within the honey bee stress response network. They connect diet quality to redox homeostasis, immunity, detoxification capacity, and ultimately, the resilience of honey bee colonies under pressure from pesticides, pathogens, and a changing climate (Contribution 10).

Within the broader apitherapy framework, honey emerges as a complementary antioxidant matrix in which phenolic compounds, enzymes, vitamins, and amino acids cooperate to buffer oxidative stress and inflammation, offering cardiometabolic, neuroprotective, immunomodulatory, and wound-healing benefits that align naturally with the health-promoting potential of propolis, pollen, and beeswax-derived preparations (Contribution 11).

In this context, comparative meta-analytic evidence indicates that the stingless bee honey tends to exhibit higher phenolic loads, stronger ferric-reducing antioxidant power, and distinctively higher moisture and free acidity than *Apis mellifera* honey, reinforcing its position as a particularly potent but still underexplored functional honey type that warrants more systematic characterization and clinical investigation (Contribution 12).
